# Using Imaging Mass Cytometry to Define Cell Identities and Interactions in Human Tissues

**DOI:** 10.3389/fphys.2021.817181

**Published:** 2021-12-22

**Authors:** Vijayakumar R. Kakade, Marlene Weiss, Lloyd G. Cantley

**Affiliations:** Section of Nephrology, Department of Internal Medicine, Yale University School of Medicine, New Haven, CT, United States

**Keywords:** imaging mass cytometry, kidney, highly multiplexed imaging, kidney-MAPPS, cell phenotype, *in situ* imaging

## Abstract

In the evolving landscape of highly multiplexed imaging techniques that can be applied to study complex cellular microenvironments, this review characterizes the use of imaging mass cytometry (IMC) to study the human kidney. We provide technical details for antibody validation, cell segmentation, and data analysis specifically tailored to human kidney samples, and elaborate on phenotyping of kidney cell types and novel insights that IMC can provide regarding pathophysiological processes in the injured or diseased kidney. This review will provide the reader with the necessary background to understand both the power and the limitations of IMC and thus support better perception of how IMC analysis can improve our understanding of human disease pathogenesis and can be integrated with other technologies such as single cell sequencing and proteomics to provide spatial context to cellular data.

## Introduction

The predominant methods for cell identification during the pathological analysis of formalin-fixed paraffin-embedded (FFPE) samples from human renal core biopsy tissues include cell morphology and immunohistochemistry or immunofluorescence (IF). The limited amount of tissue obtained from renal biopsy confines the type of analysis performed, preventing extensive analysis at the molecular and cellular level. Thus, most analyses are descriptive, with few efforts to provide quantitative information about the tubular, stromal, and nonresident cell populations in disease states (Kretzler et al., 2002; Zhang and Parikh, 2019). Single-cell RNA sequencing and single nucleus RNA sequencing have markedly increased the depth of information gained from a single biopsy, but lack the spatial information needed to determine cell proximity and cell–cell interactions (Rost et al., 2017; Cippà et al., 2018; Wu et al., 2018, 2019; Lake et al., 2019; Deleersnijder et al., 2021). The large numbers of distinct cell populations and complex cellular arrangement of the human kidney make it particularly difficult to adequately analyze without high-resolution spatial information. To provide that spatial information on such a large number of cells, several platforms for multiplexed imaging have recently been developed, including serial immunofluorescence staining, staining with DNA-barcoded antibodies (CODEX, CO-Detection by indEXing), and staining with metal-tagged antibodies [multiplexed ion beam imaging (MIBI) and imaging mass cytometry (IMC)]. For a detailed comparison of the strengths and weaknesses of these technologies, please see the comprehensive review by Baharlou et al. (2019).

## Imaging Mass Cytometry

Imaging mass cytometry (IMC) is a powerful analytical platform in which a high-resolution laser is combined with a mass cytometer that permits mass spectrometry-based, spatially preserved high-dimensional analysis of intact FFPE and frozen tissues at a resolution of 1 μm^2^ per pixel (Giesen et al., 2014; Bodenmiller, 2016; Guo et al., 2020). In IMC, a cocktail of validated antibodies against defined protein epitopes, each of which is covalently bound to a unique rare-earth metal, is hybridized on a tissue section. Pulsed ablation of 1 μm^2^ spots with a UV laser is performed, with the vaporized tissue analyzed by a mass spectrometer to identify the combination of heavy metals present in that 1 μm^2^ region. The quantity of each metal present at each 1 μm^2^ coordinate is used to reconstruct an artificial multilayer image of the initial tissue. Currently IMC can be employed for imaging up to 42 markers on a single section of tissue. The absence of endogenous signal for the heavy metals results in a very low background and improved signal-to-noise ratio, making IMC particularly appealing as compared to fluorescence-based antibody imaging (Zhang et al., 2018).

## Applications of IMC

IMC has been used to characterize malignancies, yielding information about the identity, number of, and spatial relationships between immune and resident cells (Giesen et al., 2014; Bodenmiller, 2016; Bertocchi et al., 2021; Li et al., 2021). IMC has also been applied in infectious and autoimmune diseases research and in drug profiling. The 1 μm^2^ resolution provides the ability to identify basic subcellular localization of antigens (nucleus vs. cytoplasm), while the large number of antibodies that can be simultaneously analyzed supports both cell identification and cell activation state determination. The technique can therefore be employed to identify cellular markers that provide fundamental tissue architectural layout, as well as secondary cellular responses such as protein modifications, signaling pathway activation, cell injury states, and cell proliferation. IMC allows immune cell markers to be investigated in both healthy and diseased tissues with their distribution pattern and proximity to tissue resident cells spatially mapped (Giesen et al., 2014; Catena et al., 2020; Garcia-Melchor et al., 2021; Patel et al., 2021). Analysis of functional markers generates information on disease states and can potentially be used to identify biomarkers. Importantly, the linearity of the mass spectrometry detection of the heavy metals conjugated to each antibody provides a quantitative assessment of the relative expression levels for the respective antigens, allowing researchers to detect changes not just in cell numbers and localization, but also in cell differentiation and signaling pathway activation. This quantitative information, assigned to each pixel of the generated image, supports a more objective, machine-based evaluation of the tissue that is less subject to observer bias.

IMC has been employed to characterize disease pathophysiology by providing enhanced molecular profiling of biological tissues. With that aim, several research groups have focused on the pathogenesis of type 1 diabetes. Through analysis of 1,581 islets from 12 human donors and eight type 1 diabetic patients, using a panel of antibodies targeted against 35 biomarkers, Damond et al. (2019) found that beta cell destruction is preceded by a beta cell marker loss and by recruitment of cytotoxic and helper T cells. Similarly, using a panel of 33 antibodies and quantifying pancreatic exocrine cells, islet cells, immune cells, and stromal components, Wang et al. (2019) demonstrated a dramatic change in islet architecture, endocrine cell number, and immune cell number in pancreatic sections from type 1 diabetic patients. This study also demonstrated a molecular change indicating altered cell identity and dysregulated cellular protein expression in the histopathology of type 1 diabetes. The described ability to detect and quantify protein-level alterations in cells was reflected in a separate study of neuronal changes in postmortem samples from patients with Parkinson’s disease. Using IMC analysis with a panel of antibodies targeting subunits of all five mitochondrial oxidative phosphorylation complexes, Chen et al. (2021) found a widespread decrease in expression of all complexes in Parkinson’s neurons as compared to control cases. An IMC study of tissue samples from patients with multiple sclerosis characterized a pool of immune cells including macrophages adjacent to regions of demyelinization and multiple subsets of T and B cells, delineating the cellular makeup of the immune response during exacerbations of disease (Ramaglia et al., 2019). Identifying markers to characterize the pathogenesis of different disease entities allows hypothesis generation for further research. Additionally, IMC contributes to deciphering of so far unknown targets relevant to signaling pathways involved in disease pathogenesis.

IMC has also been used to further characterize hepatitis B virus-associated liver disease and most recently, COVID-19-associated organ manifestations including lung disease (Wang et al., 2020; Zhang et al., 2020; Melms et al., 2021; Rendeiro et al., 2021), brain injury (Schwabenland et al., 2021), and small intestinal infection (Lehmann et al., 2021). Allam et al. (2021) analyzed tonsillitis samples, while at the same time introducing a new algorithm to explore spatial relationships in diverse multiplexed tissue imaging data. In these publications, the corresponding antibody panels reflected potentially relevant signaling pathways in addition to immune cell composition and cross talk.

The ability of IMC to characterize both structural cells and trafficking immune cells has been important for its use in characterizing the tumor microenvironment and treatment effects or pharmacodynamics, respectively. IMC can provide: (i) a means to investigate the cellular heterogeneity of the tumor microenvironment and understanding of cancer progression and resistance to current therapies (Ijsselsteijn et al., 2019; Elaldi et al., 2021); (ii) a basis for supporting efficacy and target engagement validation studies in drug discovery (Bouzekri et al., 2019); (iii) discovery of new biomarkers (Martinez-Morilla et al., 2021); (iv) potential novel drug targets; and (v) improved implementation of already established therapeutics (Carvajal-Hausdorf et al., 2019; Kankeu Fonkoua et al., 2021). These analyses include relevant cell populations and interactions, surrogates of cancer cell states, and immune cell markers. IMC-based analysis of the tumor microenvironment can potentially provide prognostic information predictive of disease outcome.

## Application of IMC in the Human Kidney

**Figure 1:**
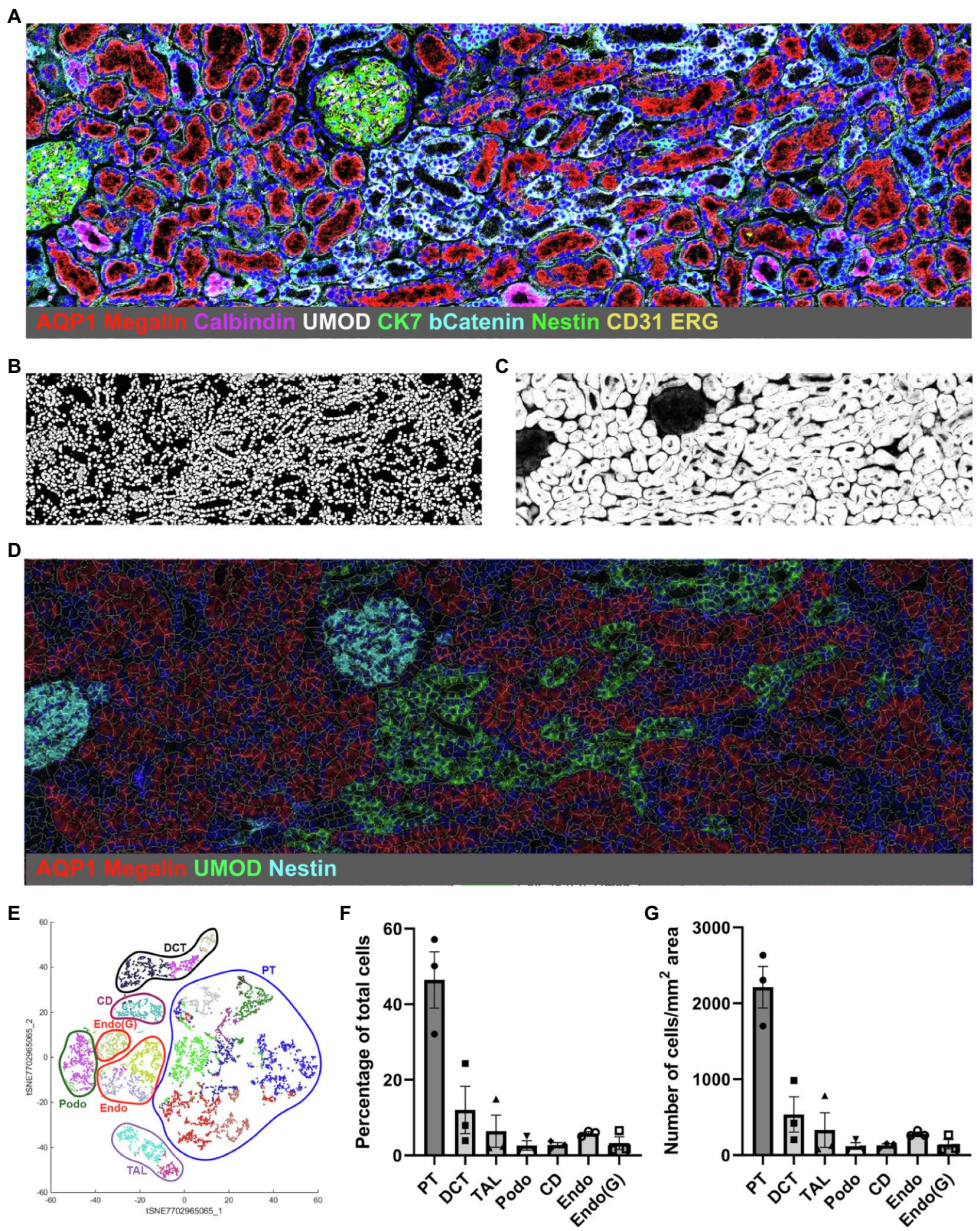
Representative Kidney-MAPPS analysis of healthy human kidney tissue for identification of resident cell populations. **(A)** Pseudocolored image of a region of interest analyzed by IMC. The depicted colors correspond to the markers listed on the image. AQP1, aquaporin-1; UMOD, uromodulin; bCatenin, β-Catenin; CK7, cytokeratin-7; and ERG, ETS-related gene. **(B,C)** Probability images for nuclei **(B)** and tubular cells **(C)**, created using the Ilastik software. **(D)** Segmentation mask generated in CellProfiler overlaid on a pseudocolored image created in HistoCAT. Highlighted markers were selected in order to represent the tissue structure analogous to **(A)** while allowing visibility of the cell segmentation. **(E)** tSNE plot showing different cell types/clusters identified through PhenoGraph analysis of three representative healthy kidney samples in HistoCAT. PT, proximal tubule; DCT, distal convoluted tubule; TAL, thick ascending limb; Podo, podocytes; CD, collecting duct; Endo, endothelial cells (non-glomerular); and Endo(G), glomerular endothelial cells. **(F,G)** Quantification of the cell types identified in **(E)** as a percentage of the total number of cells **(F)** and the number of cells per mm^2^ area **(G)**. Bars show the mean ± SD. *n* = 3.

Currently there are three publications showing IMC analysis of human kidney tissue (Singh et al., 2019; Wang et al., 2020; Avigan et al., 2021) and one publication analyzing murine kidney tissue (Brähler et al., 2018). The first analysis of kidney tissue by IMC was published by our group and characterized the cellular composition of the human kidney, comprising tubular, stromal, glomerular, and non-resident cells such as immune cells (Singh et al., 2019). The underlying hypothesis for the project was the relevance of interaction between kidney-resident and immune cells for homeostasis and disease development. The study (Singh et al., 2019) included the description of a machine learning-based analysis pipeline termed Kidney-MAPPS (Multiplexed Antibody based Profiling with Preservation of Spatial context) for unbiased pixel classification using Ilastik (Berg et al., 2019), nuclear-based cell segmentation using CellProfiler (McQuin et al., 2018), and clustering/neighborhood analysis using HistoCAT (Schapiro et al., 2017). In this approach, four different stacks of pseudocolored tiff images generated from the IMC data are subjected to pixel classification using Ilastik in order to create probability images representing nuclei, tubular, endothelial, and interstitial/glomerular cell types. The machine learning algorithm assigns a probability for the respective cell type to each pixel by differentiating between signal and background in each data set. Analysis in CellProfiler creates the sequential cell segmentation on the probability images and generates a unified mask. Cells are identified based on the presence of a nucleus as the primary object, followed by segmentation of the specific cell type (tubular, endothelial, and interstitial) to delineate the borders of each individual cell. Finally, processing in HistoCAT overlays the unified mask on the raw data from IMC, allowing multiplexed data analysis by unbiased clustering using the PhenoGraph algorithm, a nearest-neighbor-based clustering approach. Individual cell phenotypes are assigned to each cell in an unsupervised manner based on the combinatorial expression of the canonical cell identification markers, supporting classification and quantification of each cell type. Figure 1 provides a graphic overview of the steps that are used for the Kidney-MAPPS analysis pipeline.

IMC and the Kidney-MAPPS pipeline were successfully applied to 16 pathologist-verified, histopathologically normal FFPE human kidney samples (five from living donors, 11 from carefully selected, banked tumor-remote nephrectomy samples) to quantitatively characterize the cellular makeup of the reference human kidney (Singh et al., 2019). The antibodies that were employed by Singh et al. (2019) listed in Table 1, were first validated using a combination of literature search of single-cell expression data, *in vitro* cell expression, morphologic features identified by a pathologist, and cell co-localization of more than one cell-specific marker. Kidney-MAPPS was then employed to successfully generate a two-dimensional quantitative atlas of 22 distinct cell populations that were identified in the normal human kidney. In addition to well-characterized parts of the tubule, interstitium, and glomerulus, the analysis revealed several cell populations that had not yet been described. A population of megalin (low), aquaporin-1 (AQP1)^+^, aquaporin-2 (AQP2)^−^, cytokeratin-7 (CK7)^+^ tubular cells in the medulla were assigned to represent the thin descending limb of the loop of Henle. A cluster of megalin^+^, AQP1^+^, vimentin^+^ cells was suggestive of an injured, fibrotic, or regenerative cell type, while CK7^+^, AQP2^+^ cells were felt to represent principal cells. Moreover, the analysis identified cells in connecting tubular segments with morphologically larger diameter than the distal convoluted tubule (DCT) as calbindin^+^, CK7^+^, AQP2^+^ transition cells between the DCT and collecting duct. The Kidney-MAPPS protocol accurately identified, quantified, and localized ~92% of all cells in the human kidney, and quality control measures for validity and reproducibility of unsupervised analysis of data showed no significant differences in the manual vs. Kidney-MAPPS assigned phenotypes, out of over 10,000 cells scored (Singh et al., 2019). Further, the reproducibility analysis of Kidney-MAPPS yielded highly consistent quantitative results when adjacent sections of the same tissue were analyzed and showed no qualitative decrement in staining intensity in FFPE samples banked for >9 years. Tissue analysis on regions corresponding to the original region of interest with a new, identical antibody cocktail 4 months later also led to robust results (Singh et al., 2019).

**Table 1:** List of antibodies used on human kidney tissue.

Antigen	Target	Species	Clone	Metal	Dilution	Supplier	References
DNA intercalator	Nucleus	N/A	N/A	191Ir/193Ir	1:1000	Fluidigm	Singh et al., 2019; Avigan et al., 2021
Histone H3	Nucleus	Rabbit	D1H2	176Yb	1:600	Fluidigm	Singh et al., 2019
β-Catenin	Tubular epithelium	Mouse	D13A1	165Ho	1:250	Fluidigm	Avigan et al., 2021
β-Catenin	Tubular epithelium	Mouse	D10Ab	147Sm	1:500	Fluidigm	Singh et al., 2019
Megalin	Proximal tubule	Mouse	10D5.1	174Yb	1:250	EMD millipore	Avigan et al., 2021
Aquaporin-1	Proximal tubule	Rabbit	EPR11588	173Yb	1:2000	Abcam	Singh et al., 2019; Avigan et al., 2021
Uromodulin	Thick ascending limb	Rat	774056	151Eu	1:1600	R&D Systems	Singh et al., 2019; Avigan et al., 2021
Calbindin	Distal convoluted tubule	Mouse	401025	142Nd	1:400	ThermoFisher	Singh et al., 2019; Avigan et al., 2021
Aquaporin-2	Collecting duct (principal cells)	Rabbit	EPR21080	172Yb	1:300	Abcam	Singh et al., 2019; Avigan et al., 2021
Cytokeratin-7	Connecting tubule, collecting duct	Mouse	RCK105	164Dy	1:150	Fluidigm	Singh et al., 2019; Avigan et al., 2021
CD31	Endothelial cells	Mouse	JC/70A	149Sm	1:100	Abcam	Singh et al., 2019; Avigan et al., 2021
WT1	Podocytes	Rabbit	6F-H2	209Bi	1:25	ThermoFisher	Singh et al., 2019
Nestin	Podocytes	Mouse	196908	146Nd	1:200	Fluidigm	Singh et al., 2019; Avigan et al., 2021
aSMA	Smooth muscle cells	Mouse	1A4	141Pr	1:1600	Fluidigm	Singh et al., 2019; Avigan et al., 2021
Vimentin	Fibroblasts, pericytes, mesangium, podocytes	Mouse	RV202	150Nd	1:500	Abcam	Avigan et al., 2021
Vimentin	Fibroblasts, pericytes, mesangium, podocytes	Rabbit	D21H3	143Nd	1:400	Fluidigm	Singh et al., 2019
Collagen IV	Basement membrane, fibrosis	Mouse	1042	166Er	1:200	ThermoFisher	Singh et al., 2019; Avigan et al., 2021
Renin	Juxtaglomerular cells	Rabbit	EPR20693	171Yb	1:1000	Abcam	Singh et al., 2019; Avigan et al., 2021
CD68	Macrophages	Mouse	KP1	159Tb	1:600	Fluidigm	Singh et al., 2019; Avigan et al., 2021
CD66b	Granulocytes	Mouse	80H3	152Sm	1:200	Fluidigm	Singh et al., 2019; Avigan et al., 2021
CD3	T cells	Mouse	Polyclonal	170Er	1:80	Fluidigm	Singh et al., 2019; Avigan et al., 2021
CD4	Helper T cells	Rabbit	EPR6855	155Gd	1:100	Fluidigm	Singh et al., 2019; Avigan et al., 2021
CD8a	Cytotoxic T cells	Mouse	144B	162Dy	1:100	Fluidigm	Singh et al., 2019; Avigan et al., 2021
CD20	B cells	Mouse	H1	161Dy	1:100	Fluidigm	Singh et al., 2019; Avigan et al., 2021
CD11b	Granulocytes, monocytes/Macrophages	Rabbit	EPR1344	149Sm	1:50	Fluidigm	Wang et al., 2020
CD11c	Dendritic cells	Rabbit	Polyclonal	154Sm	1:50	Fluidigm	Wang et al., 2020
CD14	Monocytes/Macrophages	Rabbit	EPR3653	144Nd	1:50	Fluidigm	Wang et al., 2020
CD19	B cells	Rat	6OMP31	142Nd	1:50	Fluidigm	Wang et al., 2020
CD56	NK cells	Mouse	RNL-1	152Sm	1:50	Abcam	Wang et al., 2020
KIM-1	Kidney epithelial injury	Mouse	219211	160Gd	1:75	R&D Systems	Avigan et al., 2021
Ki-67	Proliferation	Mouse	B56	168Er	1:100	Fluidigm	Avigan et al., 2021
TNFalpha	Injury	Rabbit	Polyclonal	160Gd	1:50	Abcam	Wang et al., 2020

Our group further expanded the application of IMC and the Kidney-MAPPS analysis pipeline to analyze pre-implant kidney biopsy samples from deceased donor kidneys that were pre-identified as being at high risk for delayed graft function (DGF) following implantation as compared to living donor kidneys that were low risk for subsequent DGF (Avigan et al., 2021). The results showed that the high-risk deceased donor kidneys exhibited a highly significant reduction in tubular cells, and specifically proximal tubule cells, as compared to living donor kidneys. Of note, this decrease in tubular cell numbers did not reflect failure to successfully detect nuclei or failure to assign cell identity in the deceased donor kidney tissue, but rather a quantitative reduction of the number of tubular cells present per area analyzed. Moreover, consistent with our previous study (Singh et al., 2019), 99.5 ± 4.6% of tubular cells were correctly identified by the Kidney-MAPPS pipeline compared with manual adjudication, indicating that identification of kidney cell populations is not impaired between reference and injured tissues. Intriguingly, the study uncovered a small population of megalin(low), vimentin^+^ proximal tubular cells exclusively in deceased donor tissue that was surrounded by macrophage-rich infiltrates and co-expressed markers of injury and proliferation, kidney injury molecule 1 (KIM-1) and Ki67, respectively (Avigan et al., 2021). This is consistent with animal data showing that alternatively activated macrophages directly surround the proliferating proximal tubule S3 segment tubular cells during the week after kidney injury (Huen et al., 2015). Moreover, this cell population potentially plays a role in tubular repair because KIM-1 has been shown to be protective in models of ischemia reperfusion injury and renal transplantation (Lee et al., 2018; Al-Bataineh et al., 2021). These results demonstrate that IMC can provide spatially accurate data regarding biologically important cell–cell interactions.

IMC has also been applied to COVID-19 patient kidney samples in a study that analyzed immune cell infiltration and proinflammatory cytokine responses in specified organs of three COVID-19 patients. Compared to human healthy control kidneys, IMC analysis showed a higher count of CD11b^+^ macrophages, CD11c^+^ dendritic cells, and CD19^+^ B cells in two of the COVID-19 patient kidney samples (Wang et al., 2020). The antibodies that were used in this study are also listed in Table 1. By comparing the immune cell profiles and cytokines in the kidney to other organs, the authors suggested a tissue-specific immune response to COVID-19 that might lead to differential sensitivities of resident cell populations to the COVID-19 infection and subsequent therapies.

## Discussion

IMC is a powerful platform for high-dimensional multiplexed parallel analysis of dozens of proteins localized in intact tissue, providing single-cell resolution while preserving the spatial relationship and morphological features. Its capacity to quantify >40 markers renders IMC a useful technique in the clinical setting where tissue quantities from patient biopsies can be very limited. The ability to use banked FFPE sections without signal attenuation and with the same data outcome as freshly procured tissue also helps with the pathophysiologic study of diseases for which there are few patient samples available. An additional advantage of IMC as compared to IF is that there is no endogenous autofluorescence to negatively impact the signal-to-noise ratio. In terms of sensitivity, IMC can successfully detect a lower limit of approximately 50 copies of an epitope per 1 μm^2^ laser pulse (Hartmann and Bendall, 2020).

Limitations include the resolution that presently is at 1 μm^2^, meaning that subcellular organelles such as mitochondria, endoplasmic reticulum, and vacuoles cannot be resolved and that areas of cell overlap can make pixel classification and cell segmentation less accurate. The effect this infers on marker expression must be kept in mind when assigning a certain phenotype to a cell. In addition, the acquisition of data is time-consuming, taking 2 h to ablate 1 m^2^ area of interest, and approximately 10% of analyzed IMC regions did not yield analyzable data in the study by Singh et al. (2019).

IMC can be easily complemented by other conventional as well as highly multiplexed technologies. For example, Catena et al. (2018) developed a method to perform H&E counterstaining on IMC samples, allowing basic structural analysis of the tissue prior to IMC interrogation. By conjugating RNAscope probes to heavy metals rather than fluorophores, IMC can be used to simultaneously detect RNA and protein expression on the same tissue sample. Using this approach, unique cell identifiers that lack appropriate antibodies can be multiplexed with protein detection in single cells *in situ*, allowing correlation between transcriptional signatures and spatial context (Schulz et al., 2018). This helps gather additional information, i.e., on host–pathogen interactions when a pathogen is only detectable through RNA expression. Focusing on kidney analysis, the validated antibody panel together with the analysis pipeline and quantitative data on the makeup of cells in healthy kidney by Singh et al. (2019) provides a framework and reference cohort for future studies of diseased kidneys. The discovery of new cell types using these combined approaches will help us better understand and characterize the complexity of kidney tissue.

In summary, IMC can be applied to almost any tissue as long as the antibodies employed are appropriately validated and tested, and provides a unique ability to both detect and quantify multiple target antigens on a single archived or fresh tissue sample. We have found this to be particularly useful in the human kidney, which has historically presented many challenges to researchers due to its combination of cellular and architectural complexity. The ability to combine IMC with other tissue interrogation techniques such as single-cell RNA sequencing promises to further increase our understanding of the cellular changes that underlie kidney disease states, and thus identify appropriate interventions in a patient-specific manner.

## Author Contributions

VK and MW contributed equally to this work and wrote and edited the manuscript. LC oversaw and contributed to writing and editing the manuscript. All authors contributed to the article and approved the submitted version.

## Funding

This study was supported by DK126815 and AG067335 awards to LC and KPMP Opportunity pool award (U2CDK114886) to VK and LC. Additional support came from the German Research Foundation to MW (WE6653/1-1).

## Conflict of Interest

The authors declare that the research was conducted in the absence of any commercial or financial relationships that could be construed as a potential conflict of interest.

## Publisher’s Note

All claims expressed in this article are solely those of the authors and do not necessarily represent those of their affiliated organizations, or those of the publisher, the editors and the reviewers. Any product that may be evaluated in this article, or claim that may be made by its manufacturer, is not guaranteed or endorsed by the publisher.

## Abbreviations

IMC: Imaging mass cytometry
FFPE: Formalin-fixed paraffin-embedded
IF: Immunofluorescence
Kidney-MAPPS: Kidney-multiplexed antibody based profiling with preservation of spatial context
AQP1: Aquaporin-1
AQP2: Aquaporin-2
CK7: Cytokeratin-7
DCT: Distal convoluted tubule
KIM-1: Kidney injury molecule-1
DGF: Delayed graft function
